# Good Friday

**Published:** 1890-03-29

**Authors:** 


### WORDS OF CONSOLATION.

### GOOD FRIDAY.

Each year the church leads us by a reason of penitence and prayer, to gaze on the Great Sacrifice of our dying Lord; and once again we are drawing near that day which we call Good Friday.

Strange that the darkest hour that ever dawned on sinful earth, should have the power to comfort and console. It was for us men and for our salvation, that the Christ, the God-Man, hung on the cross, and it is good for us to go to Calvary and, kneeling at the foot of that cross, try to realise all that the Divine Sufferer bore for us. With the agony in the Garden of Gethsemane began the Passion of our Saviour. He had just eaten the Passover with His disciples, and had instituted in its stead the Sacrament of His Body and Blood, and now His soul was exceeding sorrowful even unto death. It was for love of us and for our consolation, that He was borne down by the thought of the ignominious death which awaited him; it was to teach us that He had taken all the infirmities of man, even to the terrors of death. Despised by the Romans, rejected by His own people, denied and forsaken by His disciples, He utters the sad complaint, "O ye who pass by, behold and see if there be any sorrow like unto my sorrow." But alas! there were greater and more awful sufferings yet in store; He was laden with the sins of all men. The immense burden of all the crimes which ever have been, and which ever shall be committed, was upon Him. He beheld them all distinctly; not one of them escaped Him. So overwhelming was the sight, that an angel from Heaven was sent to strengthen Him, to endure the shame and grief which possessed His soul. Look then upon Him as He hangs upon the tree, bleeding from the scourgings of the soldiers and the wounds of the nails in His hands and feet! Oh shameful sight! Midst grief and wounds and dying pain He has offered Himself freely for love of us.

See! His hands and feet are fastened;
So He makes His people free;
Not a wound whence bood is flowing
But a fount of grace shall be;
Yea the very nails which hold Him
Nail us also to the Tree.

Let us ponder on this wondrous love of the Father, which spared His Son to die for us, and on the marvellous love of the Son, who, having died for us and risen again, watches over us and our happiness. Gaze then on all these woes, this agony and bloody sweat, this cross and passion. Fall in true penitence at the feet of the Crucified One, and ask pardon for your offences. Lift the eye of faith to Him who saved you and learn the awful guilt of sin, from the fearful price which was required to pay for its expiation. Can we ever grieve enough for our share in His sufferings? Can we ever in future give way to sin and uncleanness and crucify again the Son of God? Let us flee from all wickedness and uncleanness and find refuge from our guilt in Christ's wounded side. From it flows water and blood even the Blessed Sacrament to cleanse and strengthen us, and fit us to share His heavenly glory hereafter.

Wash me and dry these bitter tears,
O let my heart no further roam;
'Tis Thine by vows, and hopes, and fears,
Long since—— O call Thy wanderer home;
To that dear home, safe in Thy wounded side,
Where only broken hearts their sin and shame may hide.

